# Retracted: Application Value of Health Education Combined with Aerobic Exercise in Nursing of Patients with Mastitis Found in Physical Examination

**DOI:** 10.1155/2023/9869642

**Published:** 2023-01-05

**Authors:** Computational Intelligence and Neuroscience

*Computational Intelligence and Neuroscience* has retracted the article titled “Application Value of Health Education Combined with Aerobic Exercise in Nursing of Patients with Mastitis Found in Physical Examination” [1] due to concerns that the peer review process has been compromised.

Following an investigation conducted by the Hindawi Research Integrity team [2], significant concerns were identified with the peer reviewers assigned to this article; the investigation has concluded that the peer review process was compromised. We therefore can no longer trust the peer review process, and the article is being retracted with the agreement of the Chief Editor.

The authors were unresponsive, and the author Xianglian Sun could not be reached by the publisher using the e-mail address provided to the publisher with the article submission.
